# A preliminary study on the early warning role of DL-malic acid in atrial fibrillation occurrence among patients with hyperuricemia

**DOI:** 10.3389/fcvm.2025.1678453

**Published:** 2025-10-20

**Authors:** Dayong Li, Huachen Jiao, Donghai Liu, Zeng Li, Shunxin Lv, Haoluo Jin, Xipeng Yan

**Affiliations:** ^1^ First Clinical Medical College, Shandong University of Traditional Chinese Medicine, Jinan, Shandong, China; ^2^ Department of Cardiology, Affiliated Hospital of Shandong University of Traditional Chinese Medicine, Jinan, Shandong, China; ^3^ School of Laboratory Animal & Shandong Laboratory Animal Center, Shandong First Medical University & Shandong Academy of Medical Sciences, Jinan, Shandong, China; ^4^ The Department of Traditional Chinese Medicine at the First People's Hospital of Jining, Jining, Shandong, China

**Keywords:** hyperuricemia, atrial fibrillation, metabolomics, biomarker, early warning indicators

## Abstract

**Background:**

There have been sufficient previous studies demonstrating that hyperuricemia (HUA) is closely associated with the occurrence of atrial fibrillation (AF).The incidence of AF in patients with hyperuricemia is higher than that in the general population. Therefore, it is meaningful to explore the serum markers of AF in the HUA population and establish early warning indicators.

**Objective:**

To preliminarily explore the correlation between HUA and AF at the metabolomics level, and to identify a group of metabolites with potential predictive power for AF that can be used for further large-scale studies.

**Methods:**

This study used untargeted metabolomics technology to detect serum metabolites of patients with AF, patients with AFHUA, and control group. Receiver operator characteristic (ROC) curve were used to analyze differential metabolites.

**Results:**

Ultimately, multiple metabolites such as L-Threonine, DL-Malic acid, L-Valine, L-Cysteine were identified as early warning markers of AF in patients with HUA. Combined ROC curve using these four metabolites between the AFHUA/Control comparison group and the AFHUA/AF comparison group showed good predictive efficacy, with Area Under the ROC Curve (AUC) = 0.923 (*P* < 0.001) in the AFHUA/Control comparison group and AUC = 0.714 (*P* < 0.001) in the AFHUA/AF comparison group. This provides an early predictive method for patients who may develop atrial fibrillation among those with hyperuricemia. And offers new approaches for the prevention and treatment of atrial fibrillation.

**Conclusion:**

This study indicates that serum metabolomics can be specifically used to predict the probability of AF occurrence in individuals with HUA, and has identified metabolites such as L-Threonine, DL-Malic acid, L-Valine, and L-Cysteine that possess potential predictive efficacy.

## Graphical Abstract

**Figure:**
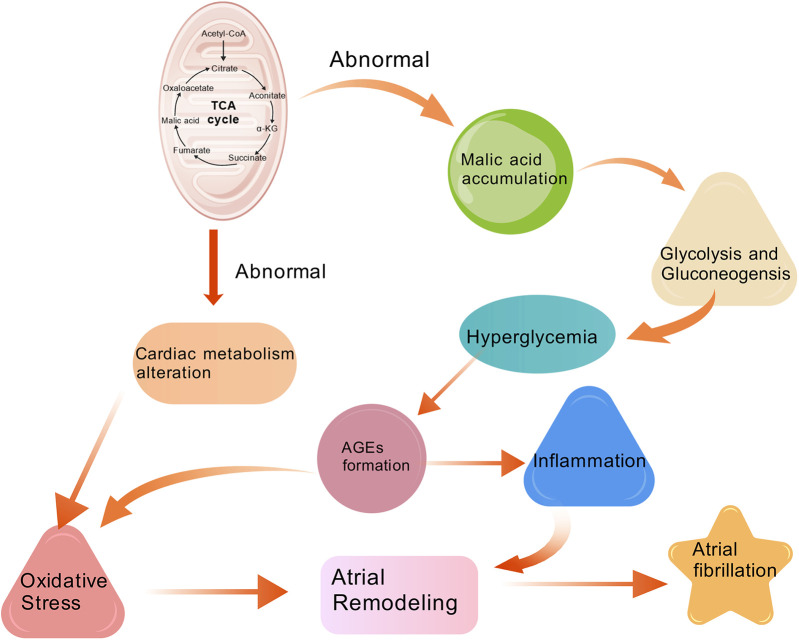
The mechanism of action of DL-Malic acid and the TCA in the development of atrial fibrillation.

## 1 Introduction

Atrial fibrillation (AF) is the most common type of arrhythmia worldwide (1). There are approximately 33.5 million people with atrial fibrillation worldwide, and the incidence rate in China is 1–2 percent (2). Atrial fibrillation can significantly affect the quality of life of patients because it is associated with serious complications such as stroke, heart failure, cognitive impairment and cardiac arrest (3). Although the clinical manifestations of atrial fibrillation are diverse. Its pathogenesis is not yet fully understood. It is currently believed to involve a combination of factors, including structural remodeling, electrical remodeling, inflammatory response, and oxidative stress (4). Hyperuricemia (HUA) is a metabolic disease caused by an imbalance of purine metabolism and uric acid production-excretion (5). HUA is defined as uric acid levels >7.0 mg/dl in men and >5.7 mg/dl in women (6). As is well-known, HUA leads to the formation and deposition of monosodium urate crystals in and around the joints, which in turn triggers gout (7). However, recent studies have found that HUA has been confirmed to be associated with the onset of multiple cardiovascular diseases. Our previous study found a significant link between HUA and the occurrence of AF. Leonardo Tamariz et al. also found that serum uric acid levels were significantly associated with new-onset atrial fibrillation in a concentration-dependent manner (8). To further investigate this association, we used non-targeted metabolomics techniques to conduct serum metabolomics analyses of different populations in three groups: the Control group, the atrial fibrillation group (AF), and the atrial fibrillation with hyperuricemia group (AFHUA), to explain this association at the metabolomics level.

## 2 Methods

**Figure 1:**
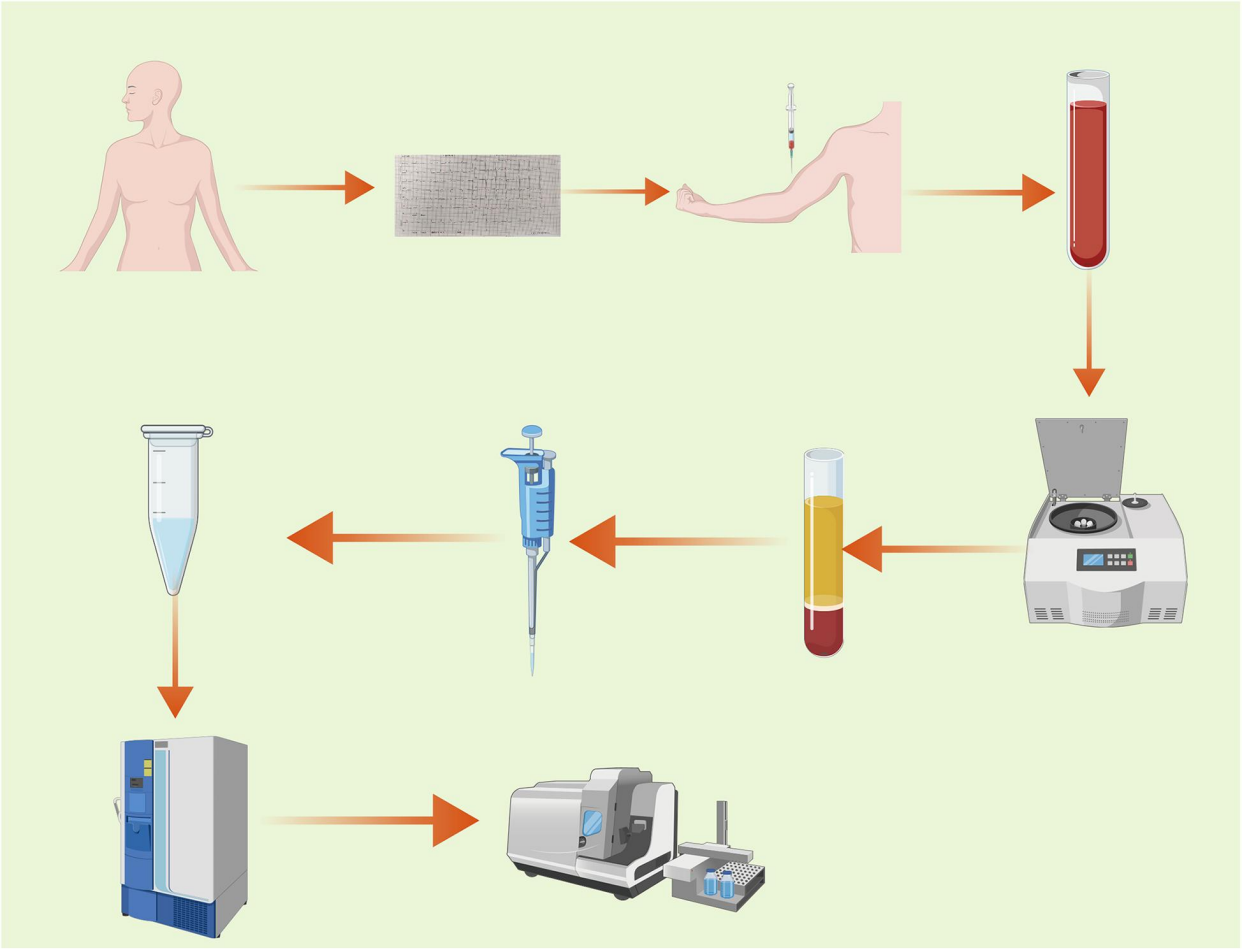
Experimental workflow diagram.

The procedure of this experiment is shown in Figure 1.

### 2.1 Study design and population

This study protocol was reviewed and approved by the Ethics Committee of the Affiliated Hospital of Shandong University of Traditional Chinese Medicine on September 13, 2023. The ethics approval number: (2023) Review No. (111) - KY. All experimental procedures were conducted in accordance with the relevant guidelines and regulations. Before the collection of blood samples, all participants provided written informed consent. Serum samples were prospectively collected from AF patients (*N* = 22), AFHUA patients (*N* = 16), and the Control group (*N* = 11) from April 2024 to January 2025 and frozen. In this experiment, the coefficient of variation is controlled at 30% (9). The preset fold change is 1.5 μ (10). Then the standard deviation *σ* = 0.3 μ, and the The expected difference between the two groups is 0.5 μ. The calculation formula is as follows:

$$n_{\mathrm{ij}}=\frac{{\left(Z_{1-\alpha /(2T)}+Z_{1-\beta }\right)}^{2}\times (\sigma_{1}^{2}+\sigma_{2}^{2})}{\sigma_{\mathrm{ij}}^{2}}$$

$$n=\max\{n_{\mathrm{ij}},\mathrm{pairs}(i,j)\}$$

*n*_ij_ represents the required sample size for pairwise comparison between the *i*-th group and the *j*-th group. *T* represents the total number of pairwise comparisons. $\sigma_{1}^{2}$, $\sigma_{2}^{2}$ represent the population variances of the *i*-th group and the *j*-th group, respectively, reflecting the dispersion of the data in both groups, $\sigma_{1}=\sigma_{2}=0.3\mu$. $\sigma_{\mathrm{ij}}$ represents the expected difference between the *i*-th group and the *j*-th group, which is taken as 0.5μ in this case. *μ* represents the mean. *α* = 0.05, *β* = 0.1. The final result is *n* = 10. In this study, the sample size for each group is greater than 10, which theoretically ensures a power greater than 0.9.

### 2.2 Sample collection

#### 2.2.1 Inclusion and exclusion criteria

##### 2.2.1.1 Inclusion criteria

1. Age 18 years or older. 2. Diagnosed with atrial fibrillation (AF) by a physician at the level of attending physician or above, according to the “Atrial Fibrillation: Current Knowledge and Treatment Recommendations - 2018.” [For the AFHUA group, in addition to being diagnosed with AF, patients must also be diagnosed with hyperuricemia (HUA) by a physician at the level of attending physician or above, according to the “Chinese Guidelines for the Diagnosis and Treatment of Hyperuricemia and Gout (2019).” The Control group must exclude both AF and HUA.) 3. Individuals who provide informed consent and sign the consent form.

##### 2.2.1.2 Exclusion criteria

1. Presence of congenital intellectual disability or diagnoses of vascular dementia, Alzheimer's disease, and other dementias. 2. Patients diagnosed with severe hepatic and renal insufficiency, acute myocardial infarction, severe heart disease, malignant tumors, cerebrovascular accidents, severe trauma, or post-major surgery. 3. Pregnant or intending-to-pregnant individuals and breastfeeding women. 4. Individuals with mental illness, alcohol abuse, or dependence on psychoactive substances.

#### 2.2.2 Patient source

All enrolled patients were inpatients from the Department of Cardiology at the Affiliated Hospital of Shandong University of Traditional Chinese Medicine between April 2024 and January 2025. The control group consisted of patients hospitalized in the Department of Cardiology for other diseases. The baseline characteristics of all participants are shown in Table 1.

**Table 1:** Participants' baseline characteristics.

Baseline characteristics	Control	AF	AFHUA	*P* value
Age, years	63.36 ± 11.07	68.55 ± 9.94	71.13 ± 11.78	0.195
Total cholesterol, mmol/L	4.33 ± 1.23	4.20 ± 1.05	3.91 ± 0.96	0.557
Low-density Lipoprotein Cholesterol, mmol/L	2.42 ± 0.86	2.39 ± 0.78	2.06 ± 0.84	0.409
BMI, kg/m^2^	27.29 ± 3.21	24.98 ± 3.29	24.61 ± 3.69	0.201
Fasting blood glucose, mmol/L	5.38 ± 1.03	5.50 ± 1.10	6.03 ± 1.97	0.547
Hypertension	8 (72.73%)	15 (68.18%)	9 (56.25%)	0.687
Coronary artery disease	7 (63.64%)	16 (72.73%)	13 (81.25%)	0.589
Smoking	0 (0.00%)	3 (13.64%)	3 (18.75%)	0.392
Drinking	1 (9.09%)	1 (4.55%)	2 (12.50%)	0.808
Male sex	5 (45.45%)	10 (45.45%)	9 (56.25%)	0.814

#### 2.2.3 Sample collection method

Blood samples were collected from all eligible subjects after fasting overnight. All blood samples were collected with the informed consent and permission of the patients. After standing for 1 h, the blood samples were centrifuged at 3,000 g for 15 min to separate the serum and blood cells. The collected serum was placed in a standard serum collection tube and stored at −80°C until the test was conducted.

### 2.3 Metabolomics analysis

#### 2.3.1 Reagents and Instruments

The instruments and reagents used in the metabolomics analysis are shown in Supplementary Table S1.

#### 2.3.2 Metabolite extraction

The experimental sample was thawed at 4℃. After thawing, the sample was vortexed for 1 min to ensure thorough mixing. Subsequently, an appropriate amount of the sample was precisely transferred into a 2 ml centrifuge tube, and 400 µl of methanol solution was added, followed by vortexing for another minute. The mixture was then centrifuged at 12,000 rpm at 4℃ for 10 min. The entire supernatant was collected and transferred to a new 2 ml centrifuge tube for concentration and drying. After that, 150 µl of a 2-chloro-L-phenylalanine (4 ppm) solution prepared with 80% methanol water was accurately added to remix the sample. The supernatant was then filtered through a 0.22 µm membrane, and the filtrate was transferred to a test bottle for Liquid Chromatography and Mass Spectrometry (LC-MS) detection (11).

#### 2.3.3 On-machine inspection

##### 2.3.3.1 Chromatographic conditions

The Liquid Chromatography (LC) analysis was performed on a Vanquish Ultra-High Performance Liquid Chromatography (UPLC) System (Thermo Fisher Scientific, USA). The High Strength Silica (HSS) T3 column was maintained at 40℃. The flow rate and injection volume were set at 0.3 ml/min and 2 μl respectively. For LC- Electrospray Ionization (ESI)(+)-MS analysis, the mobile phases consisted of (B2) 0.1% formic acid in acetonitrile (v/v) and (A2) 0.1% formic acid in water (v/v). Separation was conducted under the following gradient: 0–1 min, 8% B2; 1–8 min, 8%–98% B2; 8–10 min, 98% B2; 10–10.1 min, 98%–8% B2; 10.1–12 min, 8% B2. For LC-ESI (-)-MS analysis, the analytes were carried out with (B3) acetonitrile and (A3) ammonium formate (5 mM). Separation was conducted under the following gradient: 0–1 min, 8% B3; 1–8 min, 8%–98% B3; 8–10 min, 98% B3; 10–10.1 min, 98%–8% B3; 10.1–12 min, 8% B3 (12).

##### 2.3.3.2 Mass spectrometry conditions

Mass spectrometric (MS) detection of metabolites was performed on Q Exactive Focus (Thermo Fisher Scientific, USA) with Electrospray Ionization (ESI) ion source. Simultaneous First-Level Mass Spectrometry (MS1) and Tandem Mass Spectrometry (MS/MS) (Full MS-ddMS2 mode, data-dependent MS/MS) acquisition was used. The parameters were as follows: sheath gas pressure, 40 arb; aux gas flow, 10 arb; spray voltage, 3.50 kV and −2.50 kV for ESI(+) and ESI(−) respectively; capillary temperature, 325 ℃; MS1 range, m/z 100–1,000; MS1 resolving power, 70,000 FWHM; number of data dependant scans per cycle, 3; MS/MS resolving power, 17,500 FWHM; normalized collision energy, 30 eV; dynamic exclusion time, utomatic (9).

#### 2.3.4 Data processing and analysis

##### 2.3.4.1 Data preprocessing

Use the MSConvert tool in the Proteowizard package (v3.0.8789) to convert the original mass spectrometer off-machine file to mzXML file format (13). Peak detection, peak filtering and peak alignment were performed using the R XCMS package (14), with bw = 2, ppm = 15, peakwidth = c(5, 30), mzwid = 0.015, mzdiff = 0.01, method = “centWave” to obtain a quantitative list of metabolites. Then support vector regression correction based on quality control (QC) samples was used to eliminate systematic errors. Then the substances with a Coefficient of Variance (CV) of less than 30% (9) in the QC samples were retained for subsequent analysis.

Principal Component Analysis (PCA) and Orthogonal Partial Least Squares Discriminant Analysis (OPLS-DA) models were employed to visualize the metabolic changes among different groups. Unlike PCA, which is an unsupervised model, OPLS-DA is a supervised model that begins performing the model after pre-defined grouping information is provided (10). The metabolic profiles could be shown as a score plot, with each point representing a sample. The descriptive performance of the OPLS-DA model is determined by the parameter R-squared for Y (R²Y), which is used to describe the percentage of variance in the y-variable that is explained by the model. The closer the R^2^Y value is to 1, the more variance is explained, indicating a better fit of the model to the data. Simultaneously, the OPLS-DA model undergoes cross-validation to assess its predictive capability. The closer the model parameter Q-squared (Q^2^) is to 1, the stronger the predictive power of the model. In this experiment, the models displayed high cross-validation predictability and goodness-of-fit values. To further verify the reliability of the model, all the models were evaluated for overfitting with methods of the permutation test. The results showed that the intercept of the Q² regression line with the *Y*-axis was less than 0, indicating the reliability of the models (Supplementary Figure S1).

##### 2.3.4.2 Quality control (QC) and quality assurance (QA)

In metabolomics studies based on mass spectrometry technology, quality control (QC) is necessary to obtain reliable and high-quality metabolomics data (15). QC is an essential step to achieve stable and accurate metabolomics results. Preparation of QC Samples: A portion of the extracted samples to be tested is taken and mixed to form QC samples, which are used to correct the deviations in the analysis results of the mixed samples and the errors caused by the analytical instruments themselves. Principal component analysis (PCA) is employed to assess the reliability of the results. In the PCA score plot, the QC samples cluster in both ESI+ and ESI− modes, indicating good reproducibility of the method used. Additionally, the Relative Standard Deviation (RSD) (coefficient of variation) of potential characteristic peaks in QC samples must fall within 30% when identifying biomarkers. Therefore, quality assurance (QA) is usually performed to remove the characteristics of poor repeatability in QC samples after quality control to obtain a higher-quality data set (15). The ratio of characteristic peaks with RSD less than 30% reach about 65%, indicating the reliability and precision of the data (16) (Supplementary Figure S2).

##### 2.3.4.3 Substance identification

The metabolites were identified by accuracy mass and MS/MS data which were matched with HMDB (http://www.hmdb.ca) (17), massbank (http://www.massbank.jp/) (18), KEGG (https://www.genome.jp/kegg/) (19), LipidMaps (http://www.lipidmaps.org) (20), mzcloud (https://www.mzcloud.org) (21) and the metabolite database bulid by Panomix Biomedical Tech Co., Ltd. (Shuzhou, China). The molecular weight of metabolites was determined according to the m/z (mass-to-charge ratio) of parent ions in MS data. Molecular formula was predicted by adduct ion, and then matched with the database. At the same time, the MS/MS data from quantitative table of MS/MS data, were matched with the fragment ions and other information of each metabolite in the database, so as to realize the MS/MS identification of metabolites. A total of 1,570 metabolites were identified in this test.

### 2.4 Statistical analysis

The data were analyzed using SPSS 27.0 and GraphPad Prism 10 statistical software. All normally distributed data were expressed as mean ± SD. Nonnormally distributed data were expressed as median (interquartile range). For continuous variables, pairwise comparisons are made using the independent two-sample *t*-test. When comparing three groups, one-way analysis of variance (ANOVA) is used, followed by *post hoc* multiple comparisons tests. For categorical variables, the chi-square test or Fisher's exact test is used. The *p*-value was adjusted for multiple testing via the false-discovery rate (FDR) using the Benjamini-Hochberg method.

For the identified differential metabolites, further screening was conducted under the conditions of *P* < 0.05, value of variable importance in projection (VIP) > 1 (22), and enrichment to significant metabolic pathways (impact value >0.05, *P* < 0.05) (10). Then ROC curves were used to evaluate the predictive power of these differential metabolites for AFHUA.

## 3 Results

### 3.1 Identification of differential metabolites

**Figure 2:**
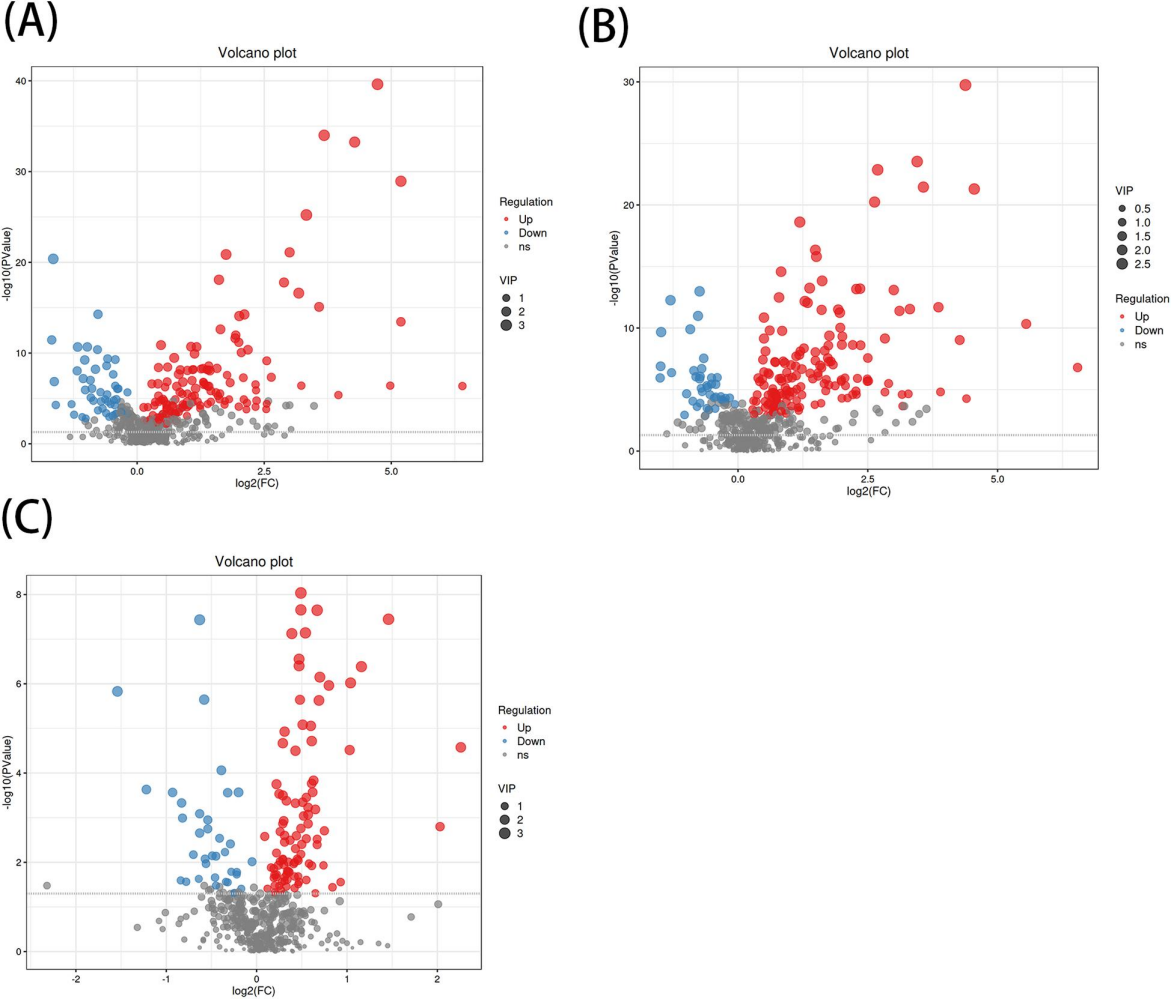
**(A)** Volcano plot of the differential metabolites in the AF/control group, with the *X*-axis being the multiple change of the log 2 transformation and the *Y*-axis being the *p*-value of the -log 10 transformation. The larger the absolute value of the *X*-axis, the greater the multiple difference in the expression of a certain metabolite between the two samples; the larger the vertical value, the more significant the differential expression. Blue indicates down-regulated significantly differentially expressed metabolites, red indicates up-regulated significantly differentially expressed metabolites, and gray dots indicate metabolites that do not meet the differential screening criteria. The size of the dots indicates the size of the VIP value. **(B)** Volcano plot of differential metabolites in the AFHUA/Control group. **(C)** Volcanic plot of differential metabolites in the AFHUA/AF group.

**Figure 3:**
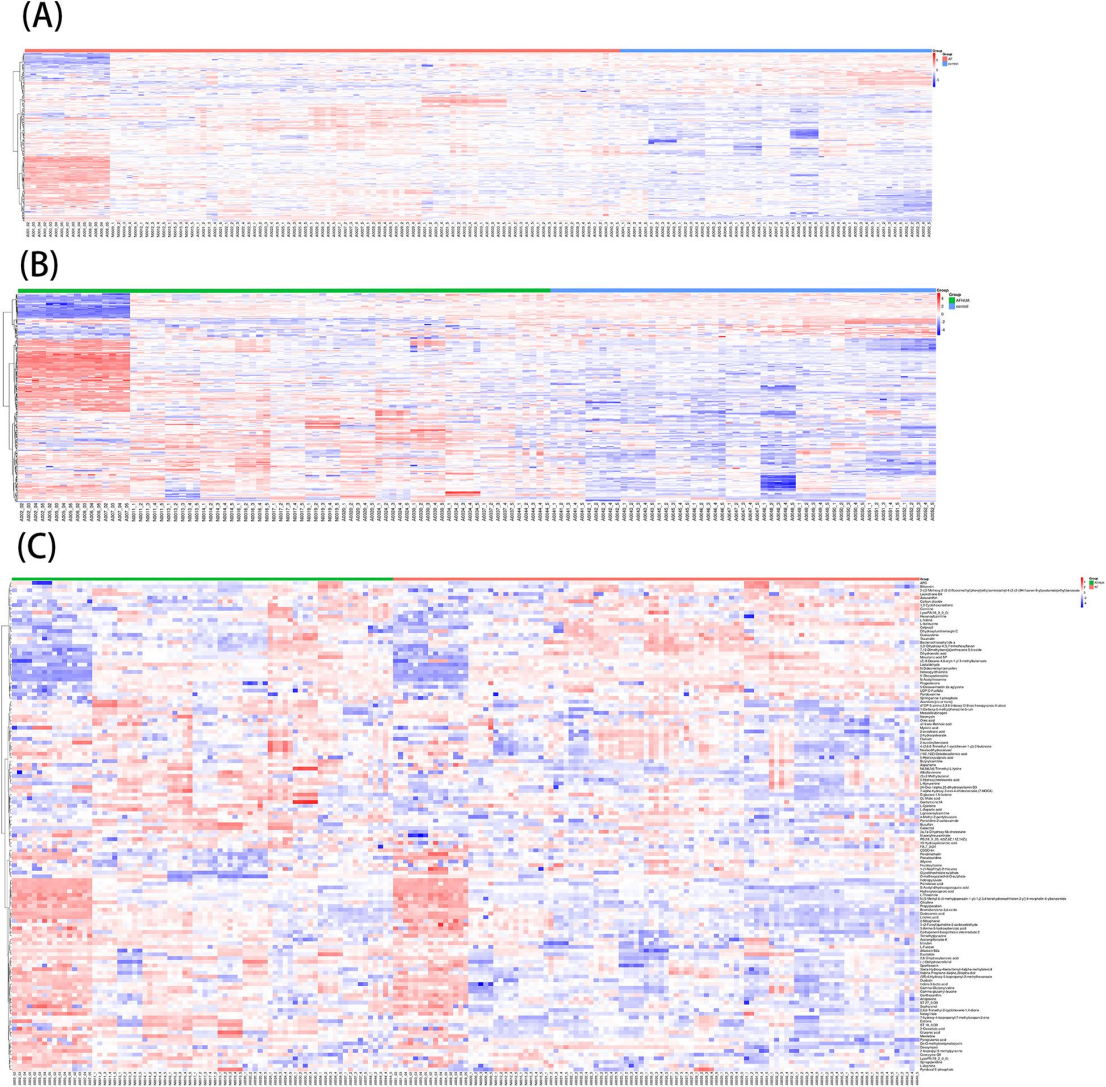
**(A)** Molecular heat map of the differential metabolites in the AF/control group. **(B)** Molecular heat map of the differential metabolites in the AFHUA/Control group. **(C)** Molecular heat map of the differential metabolites in the AFHUA/AF group.

**Figure 4:**
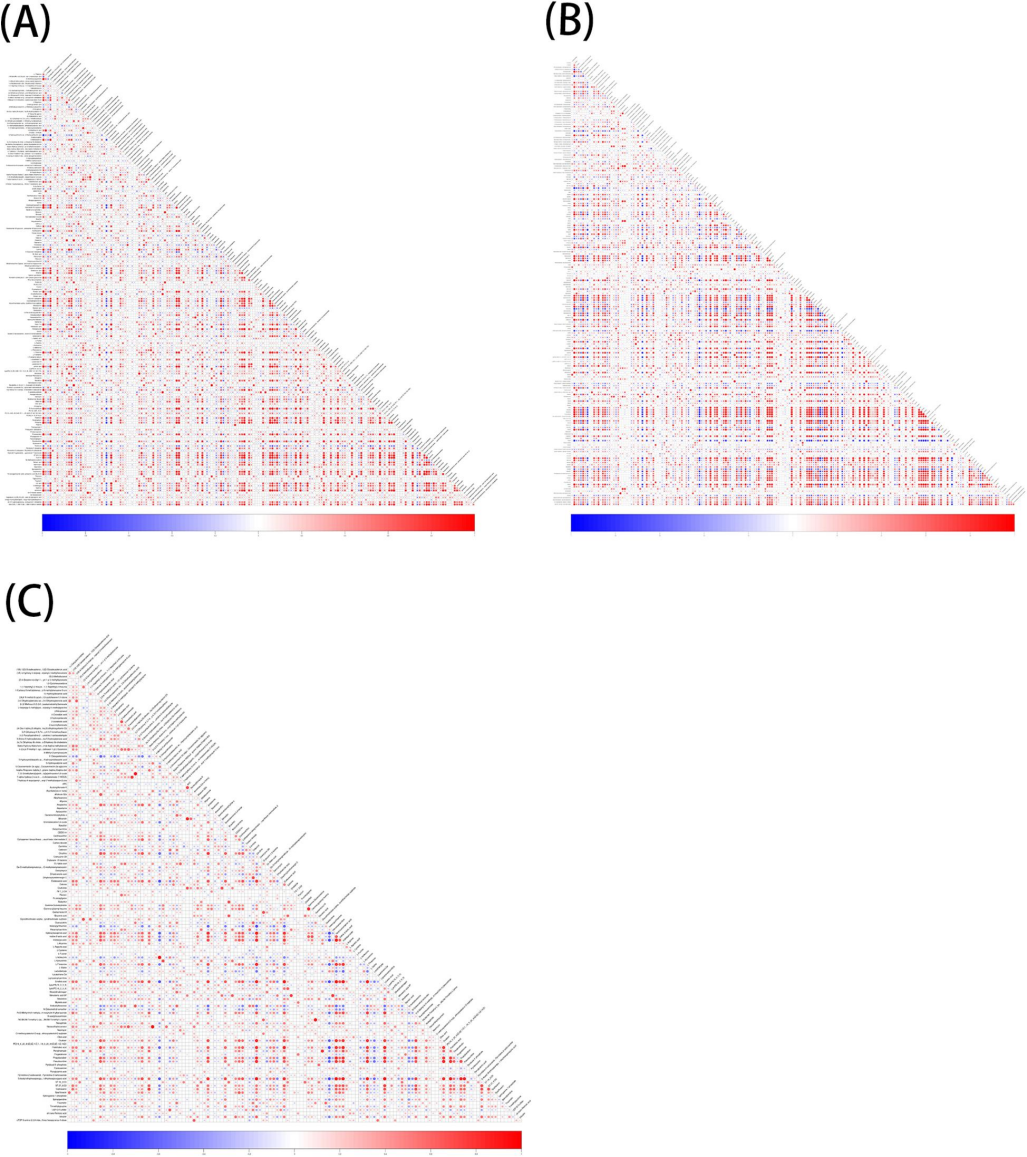
**(A)** Correlation of differential metabolites in the AF/control group, red indicates positive correlation, blue indicates negative correlation, and the darker the color, the higher the correlation. **(B)** Correlation of differential metabolites in the AFHUA/Control group. **(C)** Correlation of differential metabolites in the AFHUA/AF group.

A total of 1,570 metabolites were detected in this test. With *p* < 0.05 and VIP > 1 as the screening criteria (22). A total of 186 metabolites were significantly up-regulated or down-regulated in the AF/Control comparison group. A total of 203 metabolites were significantly up-regulated or down-regulated in the AFHUA/Control comparison group. A total of 132 metabolites were significantly up-regulated or down-regulated in the AFHUA/AF comparison group. Volcano maps (Figure 2) and differential molecular heat maps (Figure 3) show the differences of metabolites up and down in different comparison groups.For the detected differential metabolites, we plotted correlation heat maps (Figure 4) of differential metabolites to more intuitively observe the intensity of correlation among various metabolites in different comparison groups.

### 3.2 Path enrichment of differential metabolites

**Figure 5:**
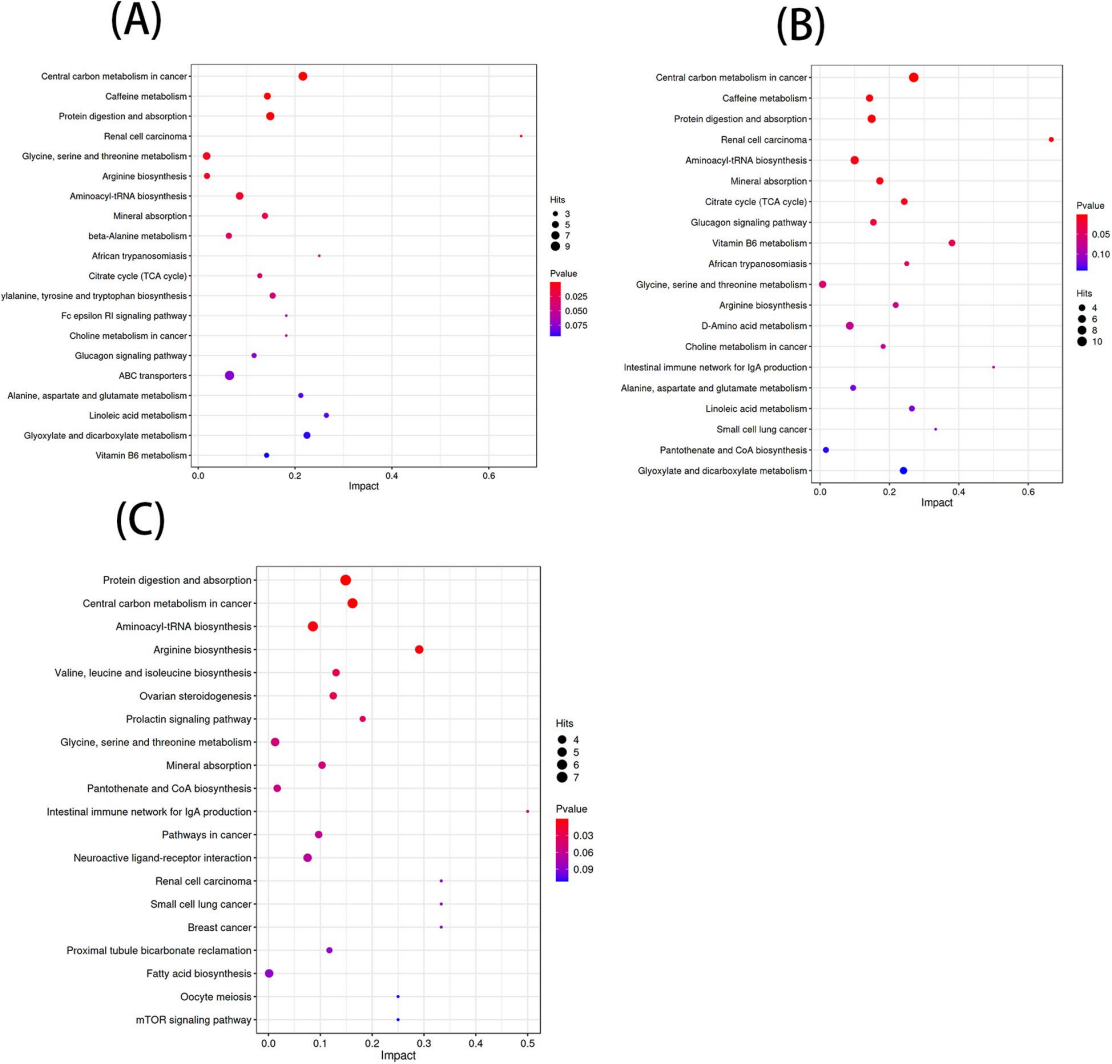
**(A)** Bubble plots of the top 20 differential metabolic pathways in the AF/control comparison groups. **(B)** Bubble plots of the top 20 differential metabolic pathways in the AFHUA/control comparison groups. Bubble plots of the top 20 differential metabolic pathways in the AFHUA/AF comparison groups.

**Figure 6:**
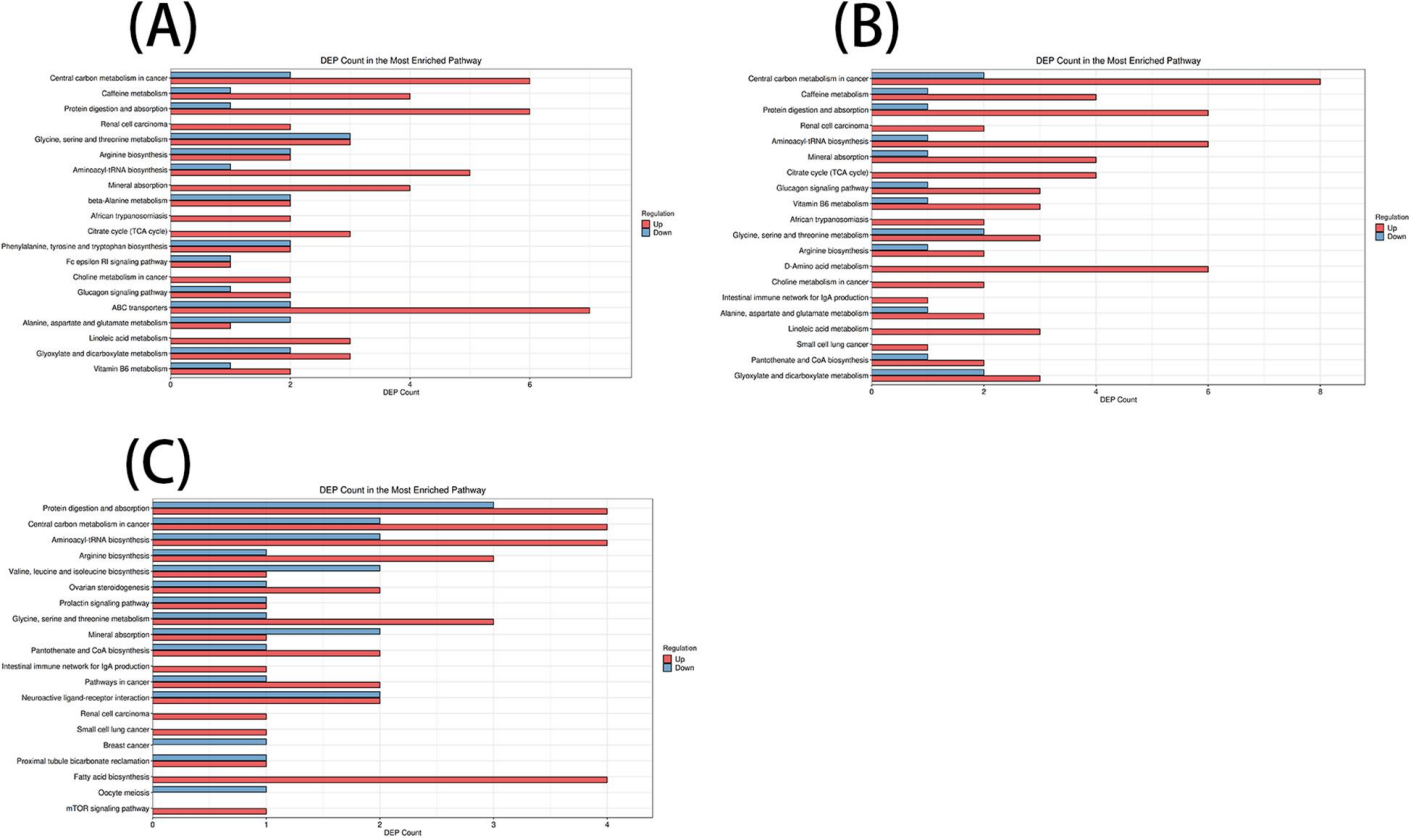
**(A)** AF/control comparison groups. **(B)** AFHUA/control comparison groups. **(C)** AFHUA/AF comparison groups. In the bubble plot, the horizontal axis represents the Impact value and the vertical axis represents the enrichment path. Point size indicates the number of metabolites corresponding to the path. The redder the color, the smaller the *P* value; the bluer the color, the higher the *p*-value. In the bar chart, the horizontal axis represents the number of metabolites, the vertical axis represents enrichment pathways, red represents up-regulation, and blue represents down-regulation.

According to the KEGG database and MetaboAnalyst software, the metabolic pathways of the differential metabolites in each comparison group were analyzed. Significant alterations in the metabolic pathways were found in three comparison groups. Bubble plots of the top 20 differential metabolic pathways in different comparison groups were plotted based on *P* values (Figure 5). Meanwhile, statistical bar graphs (Figure 6) of the number of differential metabolites enriched in each pathway were drawn. The pathways reaching the significance threshold (impact value >0.05, *P* < 0.05) were selected as the targets for further analysis. There were 10 pathways in the AF/Control comparison group. 21 differential metabolites were enriched in these 10 pathways. These differential metabolites and the differential pathways they were enriched in are shown in Supplementary Table S2. There were 10 pathways in the AFHUA/Control comparison group. 21 differential metabolites enriched in these 10 pathways. These differential metabolites and the differential pathways they enriched in are shown in Supplementary Table S3. There were 7 pathways in the AFHUA/AF comparison group. 12 differential metabolites were enriched in these 7 pathways. These differential metabolites and the differential pathways which they were enriched in are shown in Supplementary Table S4.

### 3.3 Biomarker screening

**Figure 7:**
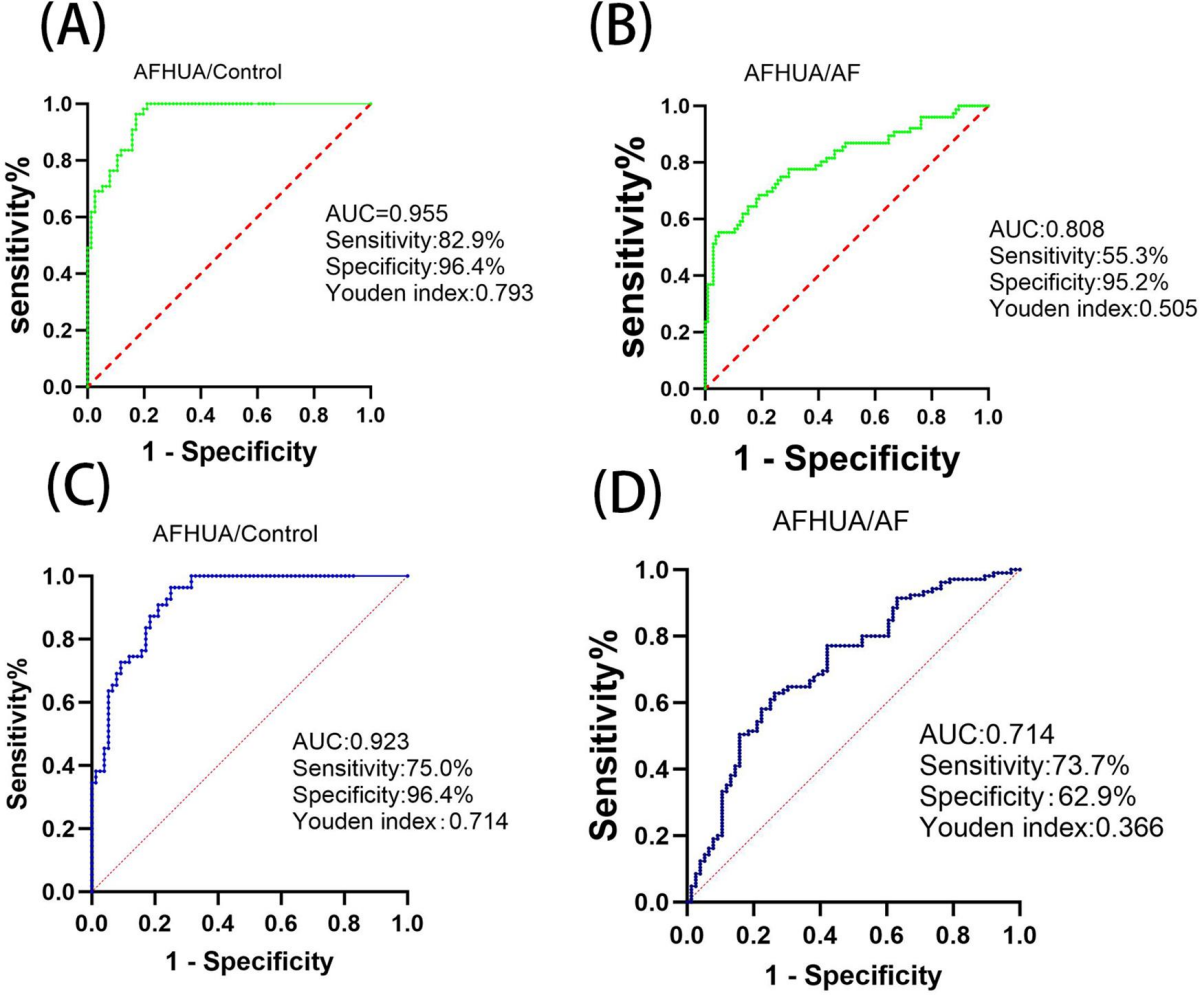
**(A)** In the AFHUA/control comparison group, the combined ROC prediction curve using L-threonine, L-histidine, L-phenylalanine, and pyridoxate. **(B)** In the AFHUA/AF comparison group, the combined ROC prediction curve using Estrone, ST 18_3; O3, L-Threonine, and L-Isoleucine. **(C)** In the AFHUA/Control comparison group, the combined ROC prediction curve using L-Threonine, DL-Malic acid, L-Valine, and L-Cysteine. **(D)** In the AFHUA/AF comparison group, the combined ROC prediction curve using L-Threonine, DL-Malic acid, L-Valine, and L-Cysteine.

ROC curve analysis was used to verify the predictive value of these differential metabolites in clinical practice. Among the 21 differential metabolites screened out from the AFHUA/Control comparison group, 19 were tested with *P* < 0.05, as shown in Table 2. Among them, L-Threonine had the best detection efficacy because its sensitivity = 0.816, specificity = 0.873, Youden index = 0.689, AUC = 0.91. To determine whether multiple metabolites can be used to jointly predict individuals in the AFHUA and Control groups. Binary logistic regression and ROC curve analysis were employed to select the four metabolites with the highest AUC values from the AFHUA/Control comparison group for the combined predict curve. The combination of L-Threonine, L-Histidine, L-Phenylalanine and Pyridoxate was found to have an AUC of 0.955 in differentiating individuals from AFHUA and Control (Figure 7A), which was significantly higher than that of any single metabolite. The 12 differential metabolites selected from the AFHUA/AF comparison group were verified by ROC curve analysis to be suitable for differential discrimination between AFHUA and AF individuals (*P* < 0.05), as shown in Table 3. Four of the 12 metabolites with the highest AUC values were selected for joint prediction by binary logistic regression and ROC curve. These four metabolites are: Estrone, ST 18_3; O3, L-Threonine, L-Isoleucine, The results showed that the AUC value of the joint prediction curve for these four metabolites reached 0.808 (Figure 7B). The detection ability was higher than that of any single metabolite.

**Table 2:** The ROC analysis between the AFHUA/Control comparison groups.

Compound	Sensitivity	Specificity	Youden index	AUC	*P*-value
L-Threonine	0.816	0.873	0.689	0.91	<0.001
L-Histidine	0.724	0.891	0.615	0.847	<0.001
L-Phenylalanine	0.816	0.727	0.543	0.838	<0.001
Pyridoxate	0.618	0.873	0.491	0.805	<0.001
Fumaric acid	0.697	0.855	0.552	0.799	<0.001
Xanthine	0.566	0.909	0.475	0.78	<0.001
Pyridoxal 5'-phosphate	0.579	0.909	0.488	0.759	<0.001
DL-Malic acid	0.553	0.927	0.480	0.743	<0.001
Pyridoxine 5'-phosphate	0.592	0.855	0.447	0.742	<0.001
L-Cysteine	0.763	0.673	0.436	0.74	<0.001
L-Methionine	0.855	0.582	0.437	0.737	<0.001
L-Valine	0.818	0.684	0.502	0.735	<0.001
Caffeine	0.566	0.818	0.384	0.731	<0.001
L-Kynurenine	0.632	0.764	0.396	0.73	<0.001
Glyoxylic acid	0.673	0.671	0.344	0.698	<0.001
Oxaloacetate	0.474	0.927	0.401	0.661	0.002
3-Methylxanthine	0.355	0.982	0.337	0.66	0.002
5-Acetylamino-6-formylamino-3-methyluracil	0.526	0.782	0.308	0.647	0.004
L-Tryptophan	0.803	0.491	0.294	0.646	0.005

**Table 3:** The ROC analysis between the AFHUA/AF comparison groups.

Compound	Sensitivity	Specificity	Youden index	AUC	*P*-value
Estrone	0.553	0.876	0.429	0.735	<0.001
ST 18_3; O3	0.566	0.886	0.452	0.726	<0.001
L-Threonine	0.592	0.752	0.344	0.687	<0.001
L-Isoleucine	0.571	0.816	0.387	0.673	<0.001
L-Aspartic acid	0.447	0.829	0.276	0.653	<0.001
Citrulline	0.566	0.762	0.328	0.652	<0.001
L-Valine	0.562	0.724	0.286	0.639	0.001
L-Arginine	0.526	0.752	0.278	0.628	0.003
Carbon dioxide	0.514	0.763	0.277	0.611	0.011
DL-Malic acid	0.289	0.924	0.213	0.591	0.038
L-Cysteine	0.368	0.829	0.197	0.589	0.041
Progesterone	0.343	0.842	0.185	0.586	0.048

**Figure 8:**
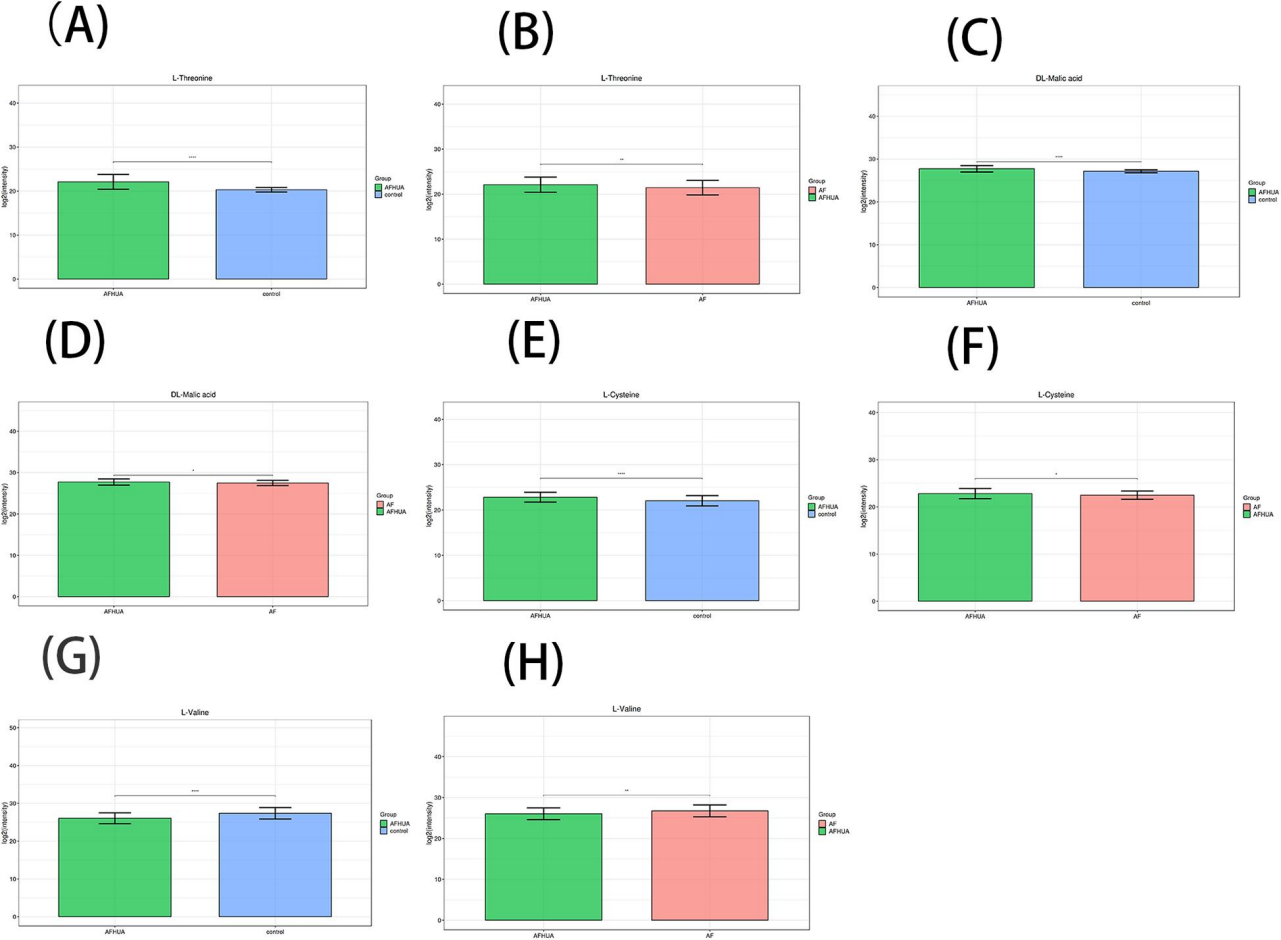
**(A)** The barplot of L-threonine levels in the AFHUA and control groups. **(B)** The barplot of L-Threonine levels in the AFHUA and AF groups. **(C)** The barplot of DL-Malic acid levels in the AFHUA and Control groups. **(D)** The barplot of DL-Malic acid levels in the AFHUA and AF groups. **(E)** The barplot of L-Cysteine levels in the AFHUA and Control groups. **(F)** The barplot of L-Cysteine levels in the AFHUA and AF groups. **(G)** The barplot of L-Valine levels in the AFHUA and Control groups. **(H)** The barplot of L-Valine levels in the AFHUA and AF groups. The Y-axis represents the concentration of the substance, while the X-axis indicates different groups.

In addition, the intersection of 19 metabolites from the AFHUA/Control comparison group and 12 metabolites from the AFHUA/AF comparison group resulted in four common metabolites. The four common metabolites are L-Threonine, DL-Malic acid, L-Valine, and L-Cysteine. Joint prediction curves using these four metabolites between the AFHUA/Control comparison group and the AFHUA/AF comparison group showed good diagnostic efficacy. With AUC = 0.923 in the AFHUA/Control comparison group (Figure 7C) and AUC = 0.714 in the AFHUA/AF comparison group (Figure 7D). This indicates that L-Threonine, DL-Malic acid, L-Cysteine and L-Valine can be used as common predictive metabolites in both the AFHUA/Control comparison group and the AFHUA/AF comparison group. The content relationship of these four metabolites in each group is as follows: the contents of L-Threonine, DL-Malic acid and L-Cysteine in each group: AFHUA > AF, AFHUA > Control, the contents of L-Valine in each group: Control > AFHUA, AF > AFHUA, the reason for this result may be due to HUA. (Figure 8) (Note: The construction of the above ROC curve models did not adjust for confounding factors.).

### 3.4 Findings

The levels of L-Threonine, DL-Malic acid, L-Valine, and L-Cysteine were significantly altered in both the AFHUA/AF and AFHUA/Control comparison groups. The combined ROC curve constructed using these four metabolites effectively distinguished samples from AFHUA and AF, with an AUC of 0.714, sensitivity of 73.7%, specificity of 62.9%, and Youden's index of 0.366. In the AFHUA/Control comparison group, the AUC was 0.923, sensitivity was 75.0%, specificity was 96.4%, and Youden's index was 0.714.

## 4 Discussion

In this study, we compared serum metabolites in AF/Control comparison group, AFHUA/Control comparison group, and AFHUA/AF comparison group. (1) Results showed that 186 metabolites were significantly up-regulated or down-regulated in the AF/Control comparison group. 203 metabolites were significantly up-regulated or down-regulated in the AFHUA/Control comparison group. 132 metabolites were significantly up-regulated or down-regulated in the AFHUA/AF comparison group. (2) Differential metabolites were screened from the AFHUA/Control comparison group, AFHUA/AF comparison group, and AF/Control comparison group on the condition that *P* < 0.05, VIP > 1 and enrichment in significant metabolic pathways. As a result, 21 differential metabolites were screened from the AFHUA/ Control comparison group. 12 differential metabolites from the AFHUA/AF comparison group. 21 differential metabolites from the AF/Control comparison group. (3) ROC curve analysis was performed on the differential metabolites selected from the AFHUA/Control comparison group and the AFHUA/AF comparison group. The results showed that 19 differential metabolites in the AFHUA/Control comparison group had Predictive value, and L-Threonine had the best Predictive efficacy. Among the AFHUA/AF comparison group, 12 metabolites have predictive value. The four metabolites with the highest AUC values in the AFHUA/Control comparison group and the AFHUA/AF comparison group were selected for combined prediction in both comparison groups. It was found that the efficacy of combined prediction in both comparison groups was better than that of any single metabolite in each group. (4) The intersection of 19 metabolites in the AFHUA/Control comparison group and 12 metabolites in the AFHUA/AF comparison group resulted in L-Threonine, DL-Malic acid, L-Cysteine, and L-Valine. Combined prediction of these four metabolites in the AFHUA/Control and AFHUA/AF comparison groups showed good predictive efficacy in both groups.

### 4.1 The role of DL-Malic acid in atrial fibrillation

DL-Malic acid, a naturally occurring acid with the chemical formula C4H6O5. It can be produced by many species and participates in the tricarboxylic acid cycle (TCA). Baseline fasting plasma levels of 2-hydroxyglutaric acid, fumaric acid and malic acid in the tricarboxylic acid cycle were found to be significantly associated with a higher risk of cardiovascular disease (23). Other studies found that intermediate metabolites of TCA such as malic acid was significantly associated with a higher incidence of AF (24). This may be because fluctuations in metabolic function can lead to the accumulation of metabolites in the TCA, including malic acid (25). Metabolic changes in the heart may increase myocardial oxidative stress (26), which adversely affects atrial structure, electrical remodeling, and myocardial function and leads to atrial fibrillation (27). Other related studies have also found that dysregulation of the TCA cycle is associated with diseases related to oxidative stress, such as cardiovascular disease (28). Earlier evidence pointed out that uric acid (UA) enters the cell through UA transporters, and intracellular UA increases the activity of xanthine oxidase (XO) and NADPH oxidase (NOX) (29). As a result, these activities promote the formation of superoxide. The NOX produces reactive oxygen species (ROS) by transferring electrons from NADPH to molecular oxygen (30). XO is deemed to be a key enzyme in UA metabolism, which is also a critical source of reactive oxygen species (ROS), free radicals responsible for oxidative damage in cardiovascular diseases (31, 32). A study that involved a histochemical staining technique based on the reduction of nitro blue tetrazolium to formazan by superoxide radical also revealed the presence of XO activity in human hearts (33). Autonomic nervous system activation can induce significant and heterogeneous changes of atrial electrophysiology and induce atrial tachyarrhythmias, including atrial tachycardia and AF (34). Recently, some researchers showed that a continuous 4 weeks inhibition of XO in infarcted rats down-regulated sympathetic innervation (35). This suggests that UA involves in sympathetic nerve activity via sympathetic innervation probably through a superoxide-dependent pathway, which eventually contributes to arrhythmia (30). The elevation of uric acid levels can increase the risk of myocardial oxidative damage by activating the renin-angiotensin system (30). An experimental test by Corry et al. found that the mRNA and intracellular protein of angiotensin II were upgraded after 48 h of UA stimulation of vascular smooth muscle cells (36). Landmesser et al. also have proved that angiotensin II induces the increased activity of NOX and XO, and eventually causes oxidative damage (37).

In addition, malic acid is an important substance in the glucagon signaling pathway. Malic acid promotes the transport of intracellular glucose to the extracellular space through glycolysis and gluconeogenesis, which raise blood glucose levels. Some studies have shown that the level of oxidative stress and atrial fibrosis were markedly increased in the subgroup of mice with wide glycemic variability and were strongly associated with enhanced AF inducibility in electrophysiological evaluation (38). Elevated extracellular blood glucose also promotes the formation of advanced glycation end products (AGEs). AGES are produced by the non-enzymatic condensation of the carbonyl group of a reducing sugar with the free amine group of a nucleic acid, protein or lipid, and then further rearrangement to produce a stable and irreversible end product (39). A large amount of evidence suggests that oxidative stress and inflammation are the central mediators of AF in the heart under metabolic stress (40–42). AGES can promote oxidative stress and inflammatory responses by altering its structure and function through binding to cell surface receptors or cross-linking to body proteins, thereby exerting pathological effects (43). KATO T reported a significant increase of atrial fibrosis and AGE-E2 expression in diabetic rats that was induced by streptozotocin, and that fibrosis in the model was partially reversed after treatment with AGES inhibitors. That suggested a causal relationship between AGE levels, atrial fibrosis and AF (44). Additional studies have shown that insulin deficiency and glycemic variability impair Phosphatidylinositol 3-kinase (PI3K) signaling pathway and bring about ionic and metabolic dysregulations capable of triggering atrial arrhythmias (45).

### 4.2 The role of L-Threonine and L-Valine in the development of atrial fibrillation

L-Threonine is one of the four configurations of threonine and it is the main biologically active form of threonine in the human body. There are few reports on the relationship between threonine and atrial fibrillation. Threonine undergoes three different metabolic pathways (46): Threonine dehydrase pathway: Threonine in the liver is catalyzed by threonine dehydrase to form α -ketobutyric acid and ammonia, and then α -ketobutyric acid is decarboxylated to form propionyl-coA, which enters the tricarboxylic acid cycle (TCA cycle). Ammonia is expelled from the body through the urea cycle. Threonine dehydrogenase pathway: Threonine is converted into 2-amino-3-ketobutyric acid under the action of threonine dehydrogenase. This intermediate is further catalyzed by 2-amino-3-ketobutyric acid coenzyme A ligase to produce acetyl-CoA and glycine. Acetyl-coA enters the tricarboxylic acid cycle to provide energy to the cell, while glycine participates in the one-carbon metabolism. Threonine aldoxylase pathway: Threonine is cleaved to produce glycine and acetaldehyde under the catalysis of threonine aldoxylase. Acetaldehyde is further oxidized to acetic acid and eventually to acetyl-coA. Acetyl-coA enters the tricarboxylic acid cycle. Studies have found (47) significant changes in L-threonine levels in the plasma of patients with atrial fibrillation compared to the Control group, and these changes have diagnostic value. Enrichment analysis has found a significant association between atrial fibrillation and the degradation pathways of threonine and 2-oxybutyric acid (α-ketobutyric acid) (*P* < 0.001, FDR *q* < 0.001). This suggests that threonine metabolism may play a significant role in the pathogenesis of atrial fibrillation. In summary, it can be found that the metabolic pathways of threonine in organisms are closely related to the tricarboxylic acid cycle. Therefor, threonine may affect the occurrence of atrial fibrillation by participating in the tricarboxylic acid cycle through its metabolites and influencing the energy metabolism of cardiomyocytes.

In this study, the content of L-Valine in the Control group and the AF group was higher than that in the AFHUA group. The ROC curve showed that the content of L-Valine was negatively correlated with the occurrence of atrial fibrillation. Katarzyna Mitrega's study also showed that L-Valine had a significant antiarrhythmic effect (48). L-Valine, as a branched-chain amino acid, is metabolized mainly in peripheral tissues such as skeletal and cardiac muscles and can protect skeletal muscles from damage after intense exercise. In addition, studies have shown that branched-chain amino acids in isolated rat hearts have enhanced post-ischemic pressure recovery, improved post-ischemic myocardial contraction and relaxation functions, and delayed ischemic myocardial contracture time (49). The ischemic protective effect on the myocardium may be the cause of L-Valine's anti-arrhythmic effect.

### 4.3 Clinical implications

The differential metabolite changes identified in this study reveal the metabolic disturbances in the development of AF, providing new insights for the clinical management and treatment of AF. On one hand, the abnormal changes in these metabolite levels can be used to infer the pathogenesis of AF, offering new theoretical underpinnings for its development and progression, and thus new strategies for the prevention and treatment of AF. On the other hand, for patients with abnormal metabolite levels, targeted metabolic regulation therapies could be considered in conjunction with traditional anticoagulant and antiarrhythmic drugs, potentially achieving a more effective comprehensive treatment plan.

### 4.4 Inference

Hyperuricemia, as a metabolic disorder, is closely associated with the occurrence of atrial fibrillation. The specific mechanism involves energy metabolism including the tricarboxylic acid cycle, glucose metabolism, amino acid metabolism, etc. By causing a series of metabolic changes, it leads to oxidative stress and inflammatory responses in the body, which in turn causes structural and electrical remodeling of cardiac cells to induce atrial fibrillation. In this study, metabolites such as L-Threonine, DL-Malic acid, and L-Valine were found to be early warning indicators of AF in patients with HUA.

### 4.5 Limitations

As a metabolomics study, the present study may have certain statistical biases due to the limited number of participants. However, as an initial exploratory study, it has preliminarily validated some metabolites with potential predictive value. Subsequently, we will conduct a larger - scale, multi - omics study to verify the findings of this exploration.

## 5 Conclusion

In this study, Metabolomics analysis revealed that the levels of metabolites such as L-threonine, DL-malic acid, and L-valine in individuals with AFHUA showed significant changes compared to the control group. The significant alterations in these metabolites not only provide new biomarkers for the early warning of AF but also offer new insights for the prevention and treatment of AF. The combined ROC curve analysis further validated the early warning potential of these metabolites, demonstrating good efficacy. In summary, this study not only elucidates the metabolic connections between hyperuricemia and AF but also provides new tools and methods for the early detection and clinical management of AF. Future research should further validate the application value of these metabolites in larger populations and explore their specific roles in the development of AF, with the aim of developing more effective strategies for the prevention and treatment of AF.

## Data Availability

The raw data supporting the conclusions of this article will be made available by the authors, without undue reservation.
