# Supplementary material for: A preliminary study on the early warning role of DL-malic acid in atrial fibrillation occurrence among patients with hyperuricemia
Source: Front Cardiovasc Med. 2025 Oct 20;12:1678453. doi: 10.3389/fcvm.2025.1678453 (PMC12580274; doi:10.3389/fcvm.2025.1678453)
Supplement: Supplementary file 1 [file Datasheet1.pdf]

Supplementary Table 1

Reagents and Instruments	Brand	Model/Purity
High-Performance Liquid Chromatography	Thermo Fisher Scientific	Vanquish
Mass Spectrometer	Thermo Fisher Scientific	Q Exactive Focus
Frozen centrifuge	Xiangyi	H1850-R
Mixer	Kylin-Bell	BE-2600
Vacuum concentrator	Eppendorf	5305
Filter membrane	Jinteng	0.22um PTFE
Methanol	Thermo Fisher Scientific	≥99.0%
Acetonitrile	Thermo Fisher Scientific	≥99.9%
2-Chlorophenylalanine	Aladdin	98%
Formic acid	Tokyo Chemical Industry	LC-MS grade
Ammonium formate	Sigma Aldrich	≥99.9%

Supplementary Table 2

Pathway_id	KEGG ID for compounds

hsa05230	C00049; C00073; C00078; C00079; C00122; C00135; C00149; C00197
hsa00232	C00048; C00385; C07481; C16357; C16365
hsa04974	C00011; C00049; C00073; C00078; C00079; C00135; C00188
hsa05211	C00122; C00149
hsa00970	C00049; C00073; C00078; C00079; C00135; C00188
hsa04978	C00073; C00078; C00079; C00188
hsa00410	C00049; C00135; C00315; C00429
hsa05143	C00078; C00328
hsa00020	C00122; C00149; C00417
hsa00400	C00078; C00079; C00251; C00944

Supplementary Table 3

**Pathway_id**	**KEGG ID for compounds**
hsa05230	C00036; C00073; C00078; C00079; C00097; C00122; C00135; C00149; C00183; C00197
hsa00232	C00048; C00385; C07481; C16357; C16365
hsa04974	C00073; C00078; C00079; C00097; C00135; C00183; C00188
hsa05211	C00122; C00149
hsa00970	C00073; C00078; C00079; C00097; C00135; C00183; C00188
hsa04978	C00073; C00078; C00079; C00183; C00188
hsa00020	C00036; C00122; C00149; C00417
hsa04922	C00036; C00122; C00149; C00197
hsa00750	C00018; C00250; C00627; C00847
hsa05143	C00078; C00328

Supplementary Table 4

**Pathway_id**	**KEGG ID for compounds**

hsa04974	C00011; C00049; C00062; C00097; C00183; C00188; C00407
hsa05230	C00049; C00062; C00097; C00149; C00183; C00407
hsa00970	C00049; C00062; C00097; C00183; C00188; C00407
hsa00220	C00011; C00049; C00062; C00327
hsa00290	C00183; C00188; C00407
hsa04913	C00410; C00468; C14209
hsa04917	C00410; C00468

Supplementary Figure 1.

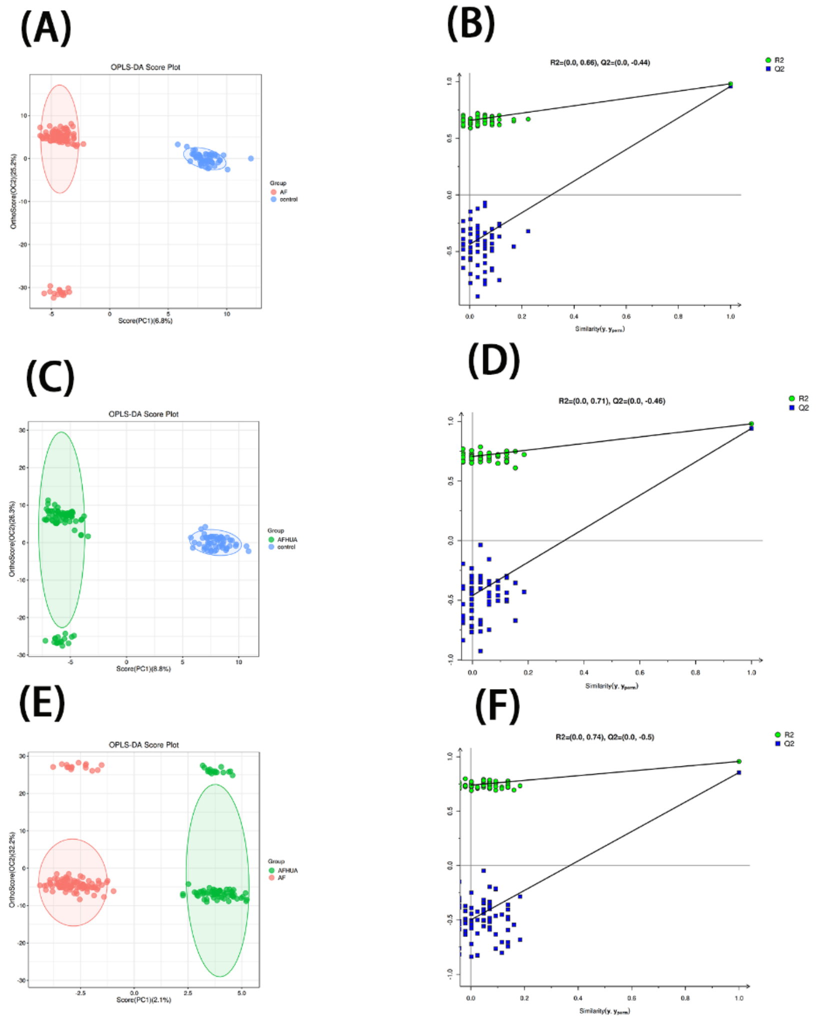

Supplementary Figure 1:(A) (B):The score plots of OPLS-DA models based on AF and Control samples and their corresponding permutation tests. (C)(D):The score plots of OPLS-DA models based on AFHUA and Control samples and their

corresponding permutation tests. (E)(F):The score plots of OPLS-DA models based on AFHUA and AF samples and their corresponding permutation tests.

Supplementary Figure 2

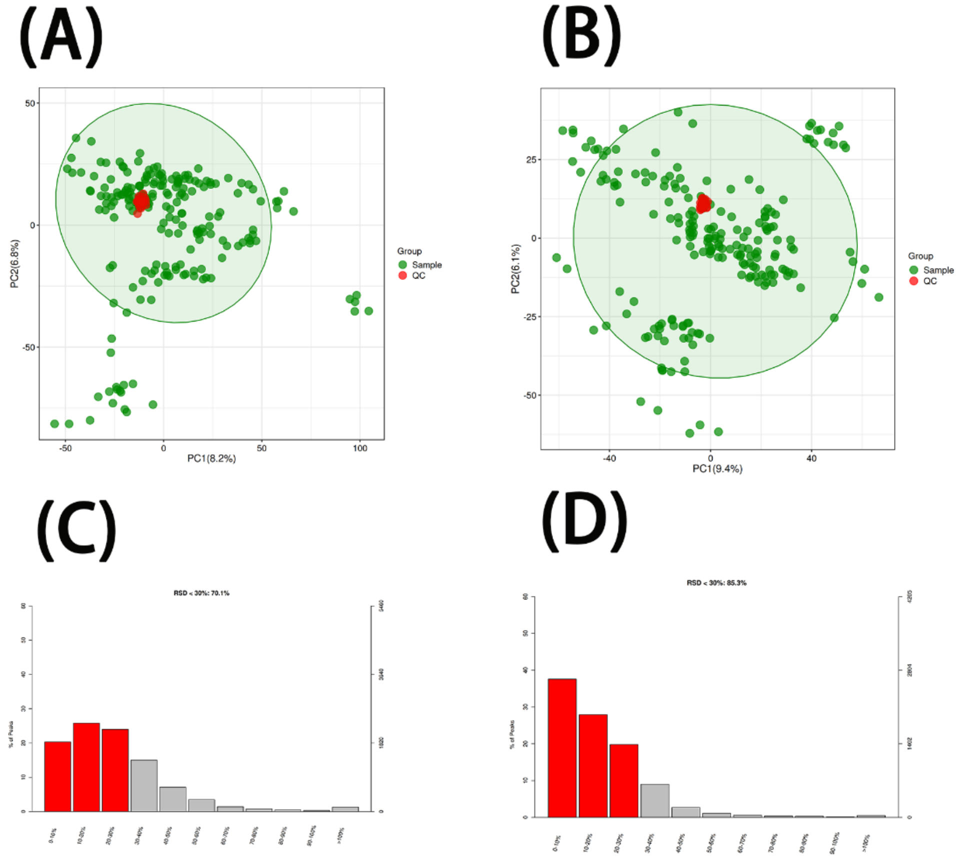

Supplementary Figure 2:A: PCA Score Plot for Quality Control (ESI+).B: PCA Score Plot for Quality Control (ESI-).C: Quality Assurance RSD Plot (ESI+).D: Quality Assurance RSD Plot (ESI-).
